# N6-methyladenosine methylation analysis of circRNAs in acquired middle ear cholesteatoma

**DOI:** 10.3389/fgene.2024.1396720

**Published:** 2024-06-24

**Authors:** Jun He, Ahmad Mahmoudi, Jacqueline Yao, Qiulin Yuan, Jinfeng Fu, Wei Liu

**Affiliations:** ^1^ Department of Otolaryngology-Head and Neck Surgery, The Second Xiangya Hospital, Central South University, Changsha, Hunan, China; ^2^ Department of Otolaryngology—Head and Neck Surgery, Stanford University School of Medicine, Stanford, CA, United States

**Keywords:** middle ear cholesteatoma, circular RNAs (circRNAs), m6A modification, microarray analysis, circRNA-miRNA-mRNA network

## Abstract

**Introduction:**

Middle ear cholesteatoma is a chronic middle ear disease characterized by severe hearing loss and adjacent bone erosion, resulting in numerous complications. This study sought to identify pathways involved in N6-methyladenosine (m6A) modification of circRNA in middle ear cholesteatoma.

**Methods:**

A m6A circRNA epitranscriptomic microarray analysis was performed in middle ear cholesteatoma tissues (n = 5) and normal post-auricular skin samples (n = 5). Bioinformatics analyses subsequently explored the biological functions (Gene Ontology, GO) and signaling pathways (Kyoto Encyclopedia of Genes and Genomes, KEGG) underlying middle ear cholesteatoma pathogenesis. Methylated RNA immunoprecipitation qPCR (MeRIP-qPCR) was performed to verify the presence of circRNAs with m6A modifications in middle ear cholesteatoma and normal skin samples.

**Results:**

Microarray analysis identified 3,755 circRNAs as significantly differentially modified by m6A methylation in middle ear cholesteatoma compared with the normal post-auricular skin. Among these, 3,742 were hypermethylated (FC ≥ 2, FDR < 0.05) and 13 were hypomethylated (FC ≤ 1/2, FDR < 0.05). GO analysis terms with the highest enrichment score were localization, cytoplasm, and ATP-dependent activity for biological processes, cellular components, and molecular functions respectively. Of the eight hypermethylated circRNA pathways, RNA degradation pathway has the highest enrichment score. Peroxisome Proliferator-Activated Receptor (PPAR) signaling pathway was hypomethylated. To validate the microarray analysis, we conducted MeRIP-qPCR to assess the methylation levels of five specific m6A-modified circRNAs: hsa_circRNA_061554, hsa_circRNA_001454, hsa_circRNA_031526, hsa_circRNA_100833, and hsa_circRNA_022382. The validation was highly consistent with the findings from the microarray analysis.

**Conclusion:**

Our study firstly presents m6A modification patterns of circRNAs in middle ear cholesteatoma. This finding suggests a direction for circRNA m6A modification research in the etiology of cholesteatoma and provides potential therapeutic targets for the treatment of middle ear cholesteatoma.

## 1 Introduction

Middle ear cholesteatoma is a pathological condition characterized by the accumulation of keratinized squamous epithelium within the middle ear and mastoid. The formation of cholesteatoma involves the growth of a cyst-like structure, which gradually expands and can lead destruction of nearby bony structures. If left untreated, cholesteatoma may result in hearing loss, vestibular dysfunction, facial paralysis, labyrinthine fistula and intracranial complications (Castle, 2018). Congenital cholesteatomas are formed by remnants of keratinizing squamous epithelium trapped in the middle ear cavity or mastoid bone during development. Meanwhile, acquired cholesteatomas result from pathological changes that cause uncontrolled growth of epithelium in the middle ear (https://www.ncbi.nlm.nih.gov/books/NBK448108/). Despite advancements in surgical techniques for cholesteatoma removal, the procedure may not restore hearing to patients’ with cholesteatoma-induced hearing loss and does not prevent disease recurrence. Given that the precise factors triggering its development, progression and recurrence remain unclear (Mor et al., 2014), further investigation is warranted to ascertain a nuanced understanding of its etiology and molecular mechanisms. N6-methyladenosine (m6A) methylation is a common and reversible RNA modification. It involves adding a methyl group to adenosine residues at the N6 position (An and Duan, 2022). Enzymes called “writers”, “erasers”, and “readers” are responsible for orchestrating the m6A modification (An and Duan, 2022). Writers add the methyl group, erasers remove it, and readers recognize and bind to the modified sites, influencing cellular processes (Shi et al., 2019; Liu et al., 2021). The dynamic nature of this modification allows regulation of mRNA stability, alternative splicing, translation efficiency, and RNA decay (Fu et al., 2014). As a result, m6A modification is a critical post-transcriptional regulatory mechanism that affects gene expression and contributes to cellular responses (Fu et al., 2014; Liu et al., 2021). 

Unlike linear RNAs, circular RNAs (circRNAs) form a closed continuous loop, increasing their stability and resistance to exonucleases (Kristensen et al., 2019). CircRNAs were initially considered as byproducts of splicing errors, whereas recent research has revealed that circRNAs have diverse and dynamic roles in cellular processes. They regulate gene expression by acting as microRNA sponges, transcriptional regulators, and potential protein translation templates (Liu and Chen, 2022). In addition to their regulatory functions, circRNAs have been associated with the pathogenesis of various proliferative diseases, including hyper-proliferation of keratinocytes in psoriasis (Chen H. et al., 2023), skeletal myoblast proliferation (Yan et al., 2022), and hypoxia-induced proliferation of vascular endothelial cells (Chen C. et al., 2023). In a previous study, we demonstrated that differentially expressed circRNAs might play an essential role in the etiopathogenesis of middle ear cholesteatoma (Xie et al., 2021). However, the role of m6A methylation modification of circRNAs in cholesteatoma etiology remains unclear.

In this study, we aim to investigate m6A modification profiles in circRNAs of middle ear cholesteatoma samples compared to normal post-auricular skin samples (Supplementary Figure S1). The findings reveal distinct patterns of m6A modification in circRNAs between middle ear cholesteatoma and normal skin samples, providing valuable insights into the pathological process of middle ear cholesteatoma.

## 2 Materials and methods

### 2.1 Individual and samples

Middle ear cholesteatoma tissue specimens were collected from five patients who underwent surgical treatment for middle ear cholesteatoma between January 2021 and December 2021. Five normal post-auricular skin samples were collected as controls. All specimens were immediately preserved in liquid nitrogen. This study was approved by The Ethics Committee of the Second Xiangya Hospital of Central South University (protocol code:2023-0171), and written informed consent was obtained from all participants.

### 2.2 RNA extraction and m6A immunoprecipitation

Cholesteatoma tissue total RNA extraction was performed using TRIzol (Invitrogen, Carlsbad, CA, United States of America). The purity and quantity of total RNA samples were assessed using a NanoDrop ND-1000, while RNA integrity was evaluated using a Bioanalyzer 2,100 or MOPS electrophoresis.

For m6A immunoprecipitation, 3–5 μg of total RNA and the m6A spike-in control combination were mixed with 300 μL of immunoprecipitation (IP) buffer (50 mM Tris-HCl, pH7.4, 150 mM NaCl, 0.1% NP40, 40 U/L RNase Inhibitor) along with 2 μg of anti-m6A rabbit polyclonal antibody. The reaction was incubated for 2 h at 4°C with head-over-tail rotation. DynabeadsTM M-280 Sheep Anti-Rabbit IgG suspension (20 µL) was blocked with 0.5% BSA at 4°C for 2 h, washed three times with 300 μL IP buffer, and then resuspended in the total RNA-antibody mixture. RNA binding to the m6A-antibody beads was achieved by head-over-tail rotation at 4°C for 2 h. Following this, the beads underwent three washes with 500 µL IP buffer and two washes with 500 µL wash buffer (50 mM Tris-HCl, pH 7.4, 50 mM NaCl, 0.1% NP40, 40 U/L RNase Inhibitor). Enriched RNA was eluted at 50°C for 1 h with 200 μL elution buffer (10 mM Tris-HCl, pH7.4, 1 mM EDTA, 0.05% SDS, 40U Proteinase K, 1 μL RNase inhibitor). RNA extraction was performed using acid phenol-chloroform, followed by ethanol precipitation.

### 2.3 Labeling and hybridization

The IP RNAs and supernatant (SUP) RNAs were subjected to the addition of an equal quantity of calibration spike-in control RNA. These RNAs were then separately amplified. SUP and IP RNA were labeled with Cy3 and Cy5, respectively, using the Arraystar Super RNA Labeling Kit (Arraystar, Rockville, MD, United States of America). The synthesized circRNAs were purified using the RNeasy Mini Kit, and their concentration and specific activity were measured using a NanoDrop ND-1000 spectrophotometer. Subsequently, Cy3 and Cy5 labeled circRNAs (2.5 g each) were combined, fragmented, and hybridized onto the m6A-mRNA & circRNA epitranscriptomic Microarray slide. The slide was incubated, washed, fixed, and then scanned using an Agilent Scanner G2505C. In this experiment, 1 μg of RNA was used for labeling. The specific activity (pmol dyes per μg circRNAs) of the labeled RNA was determined using the following formula:

$$\text{Specific Activity}=\frac{\left(\text{pmol}\;\text{per}\;\mu l\;\text{dye}\right)}{\left(\mu g\;\text{per}\;\mu l\;cRNA\right)}$$

In the case of two colors, if the yield was less than 825 ng and the specific activity was less than 8.0 pmol Cy3 or Cy5 per μg circRNA, the hybridization step was not performed. For one color, if the yield was less than 1.65 μg and the specific activity was less than 9.0 pmol Cy3 or Cy5 per μg circRNA, the hybridization step was not performed (Xie et al., 2023).

### 2.4 m6A circRNA epitranscriptomic microarray analysis

The acquired array images were analyzed using Agilent Feature Extraction software (version 11.0.1.1). Raw intensities of IP (Cy5-labeled) and Sup (Cy3-labeled) were normalized to the average of log2-scaled spike-in RNA intensities. The “m6A quantity” was calculated as the m6A modification amount based on the normalized intensities of IP (Cy5-labeled). Additionally, the m6A quantity was calculated for each transcript based on the normalized intensities of IP (Cy5-labeled), representing the m6A methylation amount.

$$m6A\text{ quantity}=IP_{Cy5\;normalized\;intensity.}$$

The m6A quantity was calculated by determining the normalized intensities of IP signals. Specifically, the raw signals of Cy5-labeled IP RNA were normalized using the average log2-scaled spike-in RNA intensities.

$${\text{IP}}_{Cy5\;normalized\;intensity}=\log2\left(IP_{Cy5\;raw}\right)-\text{ Average }\left[\log2\left({\text{IP}}_{spike-inCy5\;raw}\right)\right].$$

The differentially m6A-modified circRNAs between the Cy5-labeled IP and Cy3-labeled Sup groups were identified by applying fold change (FC) and false discovery rate (FDR) thresholds. To determine the percentage of transcripts with m6A modifications, the normalized intensities of both the Cy5-labeled IP and Cy3-labeled Sup samples were used.

### 2.5 Data analysis

The acquired array pictures were analyzed using Agilent Feature Extraction software (version 11.0.1.1). Raw intensities of IP and SUP RNAs were standardized after spike-in normalization. Probe signals with Present (P) or Marginal (M) QC flags in at least five out of ten samples were retained for further “m6A quantity” analysis. M6A methylation amount was estimated based on IP normalized intensities. Differentially methylated circRNAs between comparison groups were identified through FC and FDR thresholds (FDR < 0.05). Hierarchical clustering was performed using R software. Functional annotation and pathway analysis were conducted using GO and KEGG. A circRNA/miRNA/mRNA network was constructed based on ceRNA theory to explore circRNA mechanisms in middle ear cholesteatoma.

### 2.6 Methylated RNA immunoprecipitation with quantitative real-time PCR (MeRIP-qPCR)

Total RNA was fragmented and separated into two parts. The larger portion of fragmented RNA was subjected to immunoprecipitation with the anti-m6A antibody, while the smaller portion was saved as input RNA without immunoprecipitation. Subsequently, RT-qPCR analysis was performed on the immunoprecipitated sample RNAs and the input RNA. The PrimeScript RT reagent kit (TaKaRa, Tokyo, Japan, Cat. No. RR037A) was used to generate cDNA, and the Arraystar SYBR^®^ Green qPCR Master Mix (Arraystar, Rockville, MD, United States of America, Cat. No. ASMR-006-5) was used for quantification. Three replicates were tested for each sample. The data were normalized to the input RNA. CTA850 (positive control) and CTA650 (negative control) were used for qPCR normalization. The specific amplification primer pairs are listed in Table 1.

**TABLE 1:** Primers sequences of the circRNAs for MeRIP-qPCR validation.

CircRNA	Forward/reverse primers	Tm (°C)	Product length (bp)	Methylation
hsa_circRNA_031526	F:5′ ATAAAGTCCCCTGAGGATGTCTAC 3′ R:5′ TGGTGTCTCTGAAGTGAAGGCT 3′	60	91	Hyper
hsa_circRNA_061554	F:5′ AAGATTCAGACCTCCCATAGTTC 3′ R:5′ TGATTTTCCAGGAGAGAGATGAGA 3′	60	130	Hyper
hsa_circRNA_001454	F:5′ CAGGACATACCTTTCGTAGAGTT 3′ R:5′ TTGTTTTGCTGTGAAGAGGAG 3′	60	135	Hyper
hsa_circRNA_100833	F:5′ ATGGCAGCCCATCGAGGAT 3′ R:5′ CCAGTTCACCAATCAGCAGG 3′	60	92	Hypo
hsa_circRNA_022382	F:5′ ATGGCTGGATTCCTACCCT 3′ R:5′ TTGAGTTCTTGCCGTGGTC 3′	60	267	Hypo

### 2.7 Statistical analysis

Differentially m6A-modified circRNAs between middle ear cholesteatoma and normal skin group were identified based on FC and false discovery rate (FC ≥ 2 or ≤1/2, FDR < 0.05) thresholds using R software for hierarchical clustering. GO analysis and Fisher’s exact test for pathway analysis was conducted in the R environment. Statistical significance was defined as *p* < 0.05.

## 3 Results

### 3.1 Vast majority of m6A-modified circRNAs are hypermethylated

**FIGURE 1:**
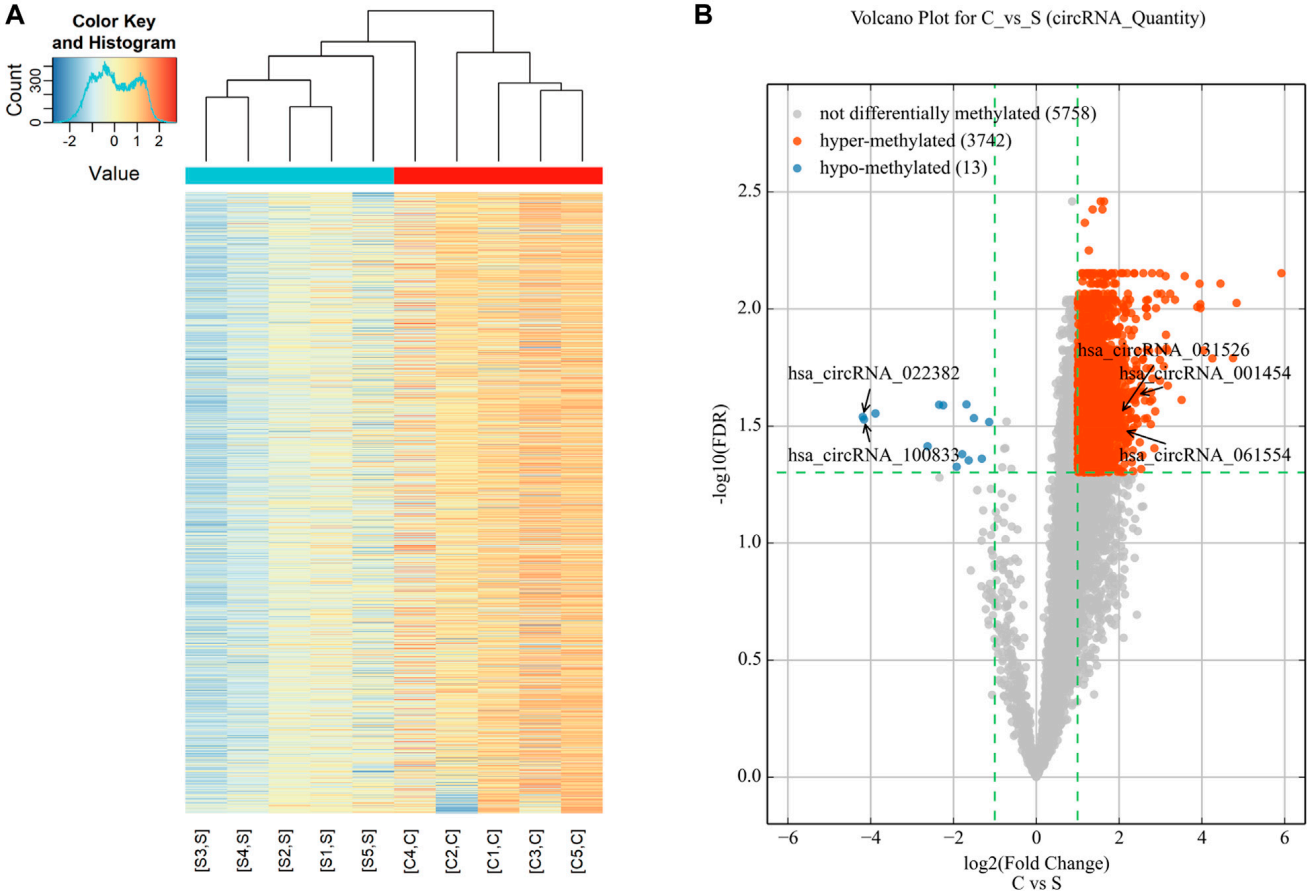
Expression profiles of significantly altered circRNAs in middle ear cholesteatoma (C) and normal skin (S). **(A)** The heatmap reveals distinct circRNA methylation patterns between middle ear cholesteatoma and normal skin. The red and blue lines represent middle ear cholesteatoma (C) and normal skin (S), respectively. Each group includes five samples. **(B)** CircRNAs differentially modified by m6A methylation in middle ear cholesteatoma and normal post-auricular skin, according to the volcano diagram analysis. The red and blue dots represent significantly hyper- and hypomethylated circRNAs (fold change ≥2 or 1/2, FDR < 0.05).

Microarray analysis was used to reveal the m6A modification patterns of circRNAs (Figure 1). Hierarchical clustering identified distinct circRNA m6A alteration patterns between the cholesteatoma and normal skin groups (Figure 1A). 3,755 circRNAs were significantly differentially modified by m6A methylation in middle ear cholesteatoma compared to normal post-auricular skin. Among these, 3,742 were hypermethylated (FC ≥ 2, FDR < 0.05) and 13 were hypomethylated (FC ≤ 1/2, FDR < 0.05) (Figure 1B).

### 3.2 Eight circRNA biological pathways hypermethylated in middle ear cholesteatoma

**FIGURE 2:**
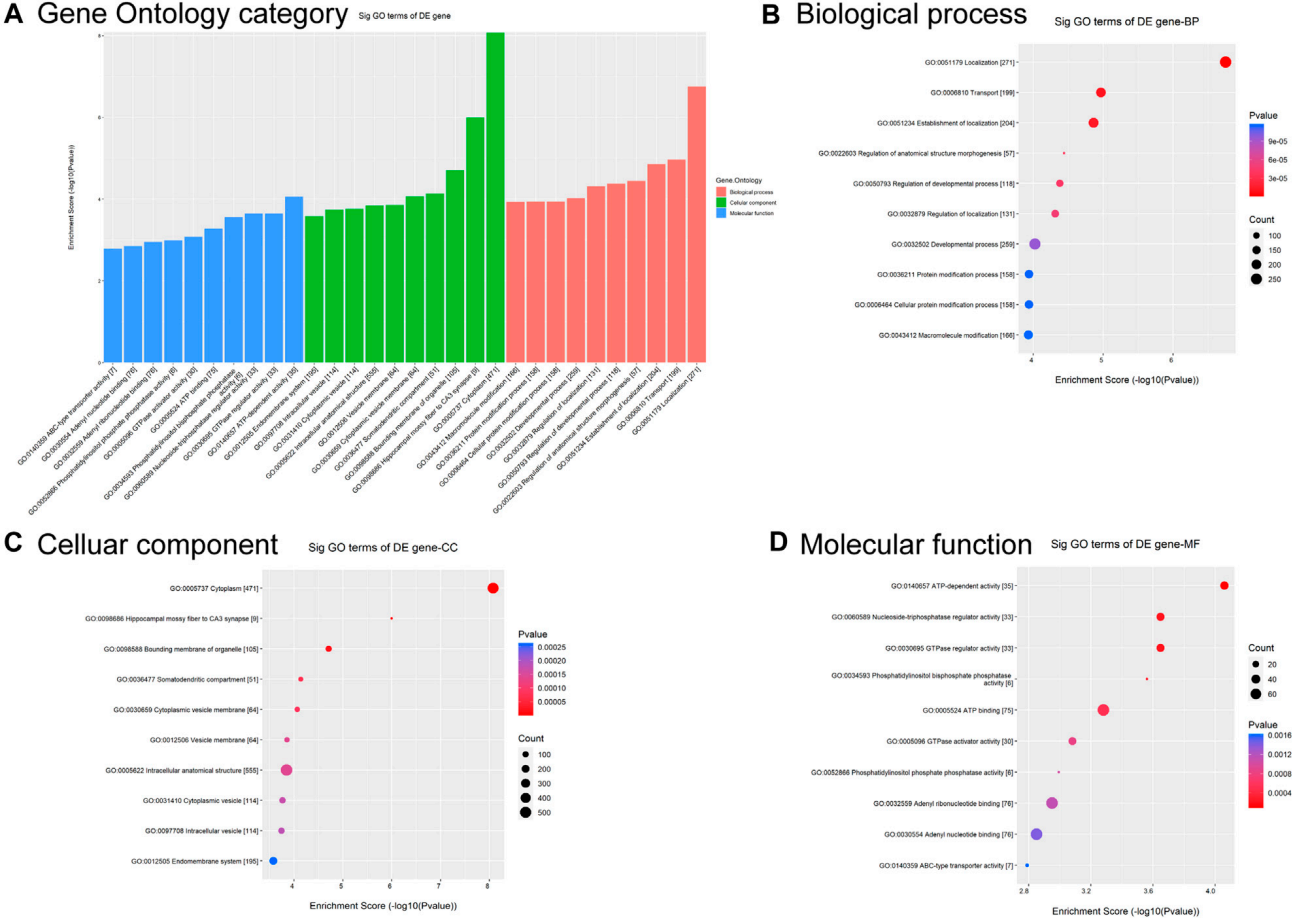
GO analysis of circRNAs differentially modified by m6A methylation. **(A)** Top 10 significantly differentially modified circRNA-enriched GO items. **(B)** Biological process. **(C)** Cellular component. **(D)** Molecular function.

To predict functional annotations and related pathways, we conducted Gene Otology (GO) enrichment and Kyoto Encyclopedia of Genes and Genomes (KEGG) pathway analyses. The GO enrichment analysis of differentially modified circRNAs included biological processes (BP), cellular components (CC), and molecular functions (MF) (Figure 2A). Among BP, the term with the highest enrichment score was localization (Figure 2B). For CC, the term with the highest enrichment score was cytoplasm (Figure 2C). The term with the highest score for MF was ATP-dependent activity (Figure 2D).

**FIGURE 3:**
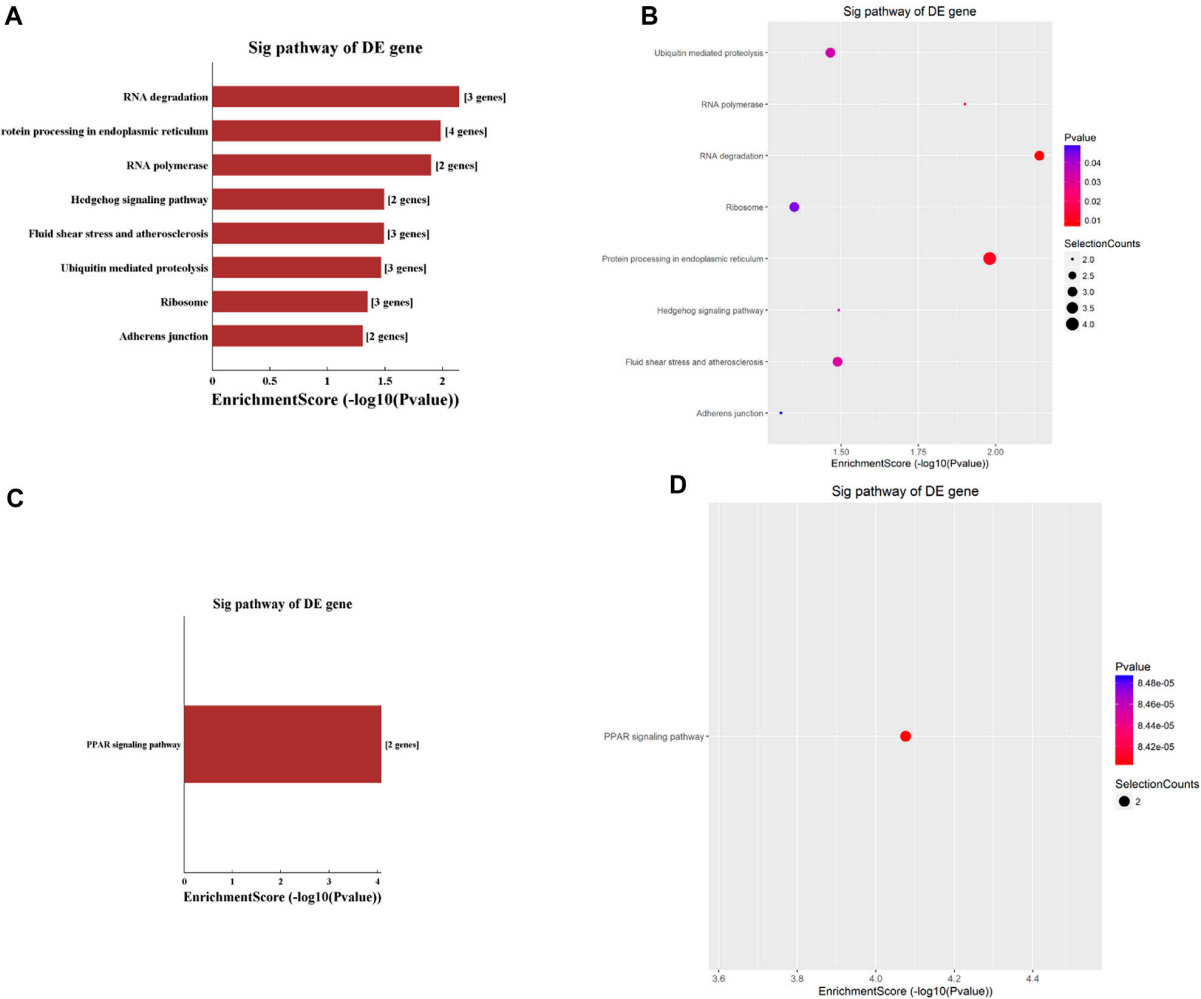
Pathway analysis based on the KEGG was conducted to explore the functional implications of circRNAs differentially modified by m6A methylation. **(A,B)** highlight pathways enriched with significantly hypermethylated circRNAs, while **(C,D)** depict the pathway enriched with significantly hypomethylated circRNAs.

The KEGG analyses identified nine pathways that circRNAs involved, with eight of them being hypermethylated. These eight pathways included the RNA degradation pathway, protein processing in the endoplasmic reticulum, RNA polymerase, hedgehog signaling pathway, fluid shear stress and atherosclerosis, ubiquitin-mediated proteolysis, ribosome, and adherens junction pathway (Figures 3A, B). Among these eight pathways, the RNA degradation pathway had the highest enrichment score. The only hypomethylated circRNA pathway was the Peroxisome Proliferator-Activated Receptor (PPAR) signaling pathway (Figures 3C, D).

### 3.3 MeRIP-qPCR validation of differentially m6A-modified circRNAs consistent with microarray analysis results

**FIGURE 4:**
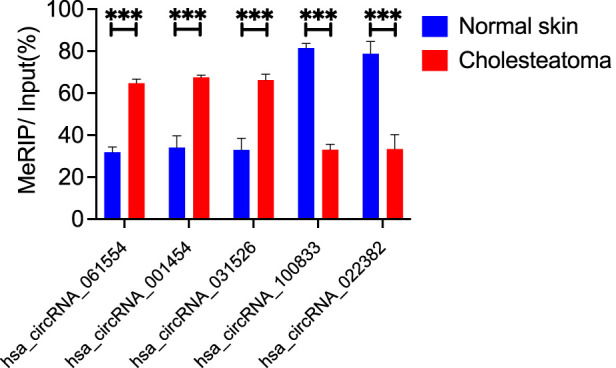
m6A methylation levels of circRNAs verified by MeRIP-qPCR. We selected five differentially modified circRNAs with m6A methylation. Hsa_circRNA_061554, hsa_circRNA_001454, and hsa_circRNA_031526 were significantly hypermethylated in cholesteatoma, while the m6A methylation levels of hsa_circRNA_100833 and hsa_circRNA_022382 were significantly downregulated. Data are presented as mean ± SEM. ***p < 0.001, unpaired *t*-test.

To validate the microarray analysis, we conducted MeRIP-qPCR to assess the m6A methylation levels. We prioritized selected hypermethylated circRNAs with larger FC values and smaller *p*-values and hypomethylated circRNAs with both smaller FC and *p*-values. Based on above criterion, we selected five specific circRNAs:hsa_circRNA_061554, hsa_circRNA_001454, hsa_circRNA_031526, hsa_circRNA_100833, and hsa_circRNA_022382. In the middle ear cholesteatoma group, we observed significantly increased m6A methylation levels in hsa_circRNA_061554, hsa_circRNA_001454, and hsa_circRNA_031526, while hsa_circRNA_100833 and hsa_circRNA_022382 showed significantly decreased m6A methylation levels (*p* < 0.05). These results from the MeRIP-qPCR experiment were highly consistent with the findings from the microarray analysis (Figure 4).

### 3.4 Construction and analysis of the circRNA–miRNA–mRNA network

**FIGURE 5:**
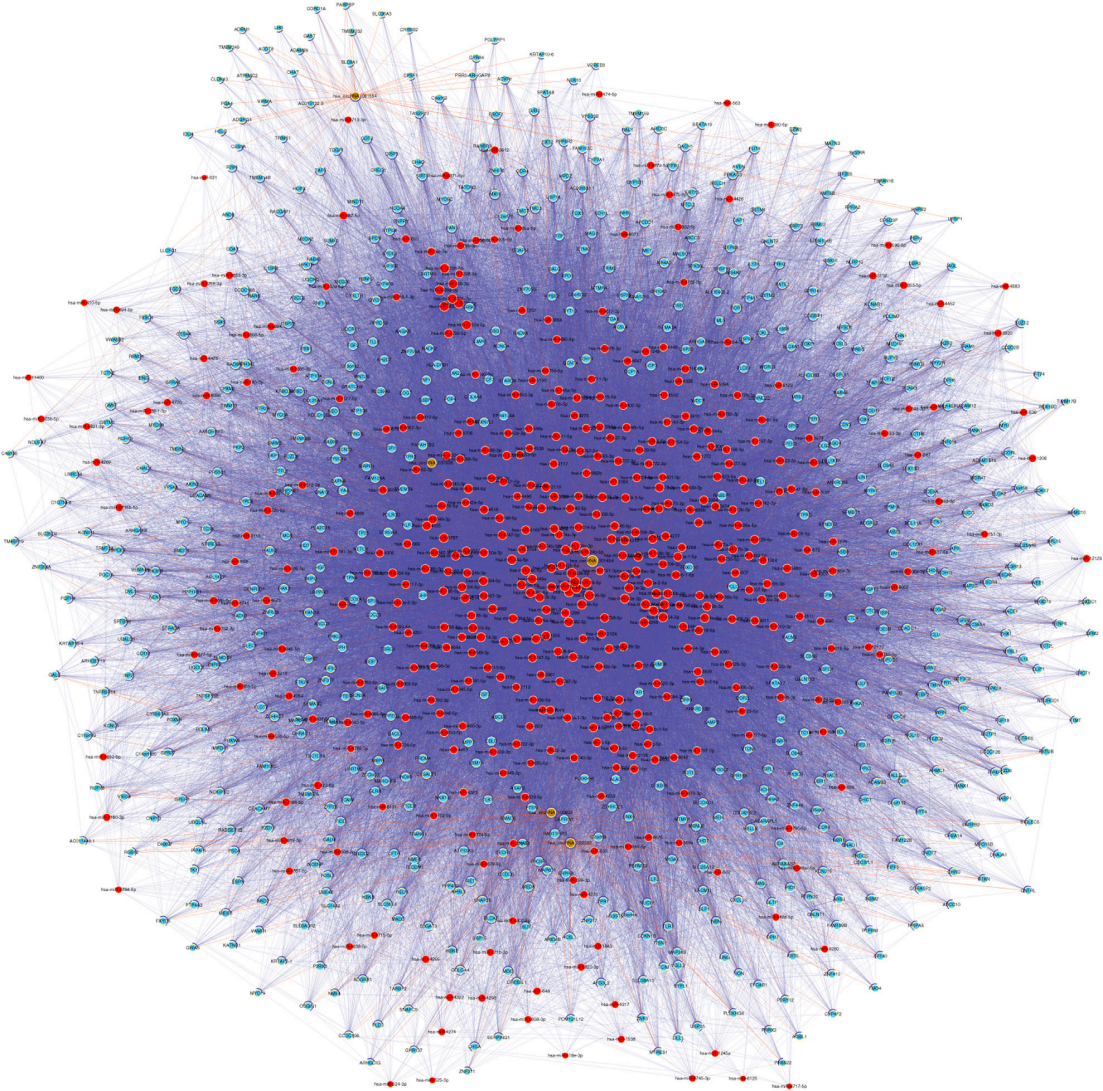
According to the ceRNA hypothesis, a network consisting of circRNA-miRNA-mRNA was established. This network is built upon five validated m6A-modified circRNAs. In the network, microRNAs, protein coding RNAs, noncoding RNAs, and circRNAs are represented by nodes colored in red, light blue, light green, and brown respectively. Directed relationships are depicted by edges with a T-shaped arrow, while undirected relationships are shown by edges without arrows.

CeRNA (competing endogenous RNA) is a regulatory mechanism in which different RNA transcripts, such as messenger RNAs (mRNAs), long non-coding RNAs (lncRNAs), and circRNAs, compete for binding to shared microRNAs (miRNAs) (Tay et al., 2014). This competition occurs through miRNA response elements (MREs) present in the transcripts, which act as molecular sponges or decoys for miRNAs. By sequestering miRNAs, ceRNAs can indirectly regulate the expression of other transcripts targeted by the same miRNAs, leading to a complex regulatory network. The ceRNA hypothesis has gained significant attention in recent years due to its potential implications in various biological processes, including cancer development (Kristensen et al., 2022), neurological disorders (Floris et al., 2017), and other diseases. However, the precise mechanisms and functional relevance of ceRNA networks in middle ear cholesteatoma are still under investigation. Based on the five validated circRNAs with significantly altered m6A methylation levels (FC ≥ 3 or ≤ 1/3, *p* < 0.05), a circRNA-miRNA-mRNA ceRNA network was constructed to investigate whether circRNAs possessed ceRNA potential in the etiology of cholesteatoma. The network consisted of five circRNAs, 447 microRNAs, and 710 protein coding RNAs. There were also 48,128 directed and 748 undirected edges (Figure 5; Supplementary Figure S2).

## 4 Discussion

Middle ear cholesteatoma is a condition that can result in a range of serious complications, both intra- and extra-cranially (Kuo et al., 2021). Despite its clinical significance, the precise pathogenesis of this condition remains poorly understood, and procedural intervention currently represents the sole therapeutic option. Furthermore, cholesteatoma displays a notable propensity for recurrence, necessitating repeated surgical procedures. m6A methylation has been documented to regulate post-transcriptional gene expression and exhibits the potential to exert an influence on the progression of various diseases (Jiang et al., 2021). However, the role of circRNA m6A modification in middle ear cholesteatoma pathogenesis has not been thoroughly studied. To our knowledge, this is the first study to investigate m6A modification of circRNAs in middle ear cholesteatoma.

This study employed m6A-modified circRNA microarray analysis and MeRIP-qPCR validation to explore the RNA epigenetic profiles of m6A-modified circRNAs in middle ear cholesteatomas. Comparative analysis with normal post-auricular skin samples revealed 3,755 circRNAs in middle ear cholesteatoma patients with m6A methylation differences. Specifically, among these 3,755 circRNAs, 3,742 exhibited hypermethylation while 13 showed hypomethylation (Figure 1). Accumulating evidence has shown that aberrant m6A modification of circRNAs contribute to the development of various diseases, including hepatocellular carcinoma (Chen et al., 2022), innate immune system inhibition (Chen et al., 2019), osteosarcoma (Liu et al., 2023) and age-related cataract progression (Li et al., 2023). These suggest that the m6A-modification patterns of circRNA found in our study may play a significant role in the pathogenesis of middle ear cholesteatoma.

GO and KEGG pathway analyses were used to predict the potential biological functions and identify the signaling pathways enriched with differentially m6A-modified circRNAs. GO analysis showed that differentially m6A-modified circRNAs were associated with localization, cytoplasm, and ATP-dependent activity (Figure 2). The role of m6A-modified circRNA in metabolism is corroborated by previous findings that this regulatory axis is involved in mitochondrial oxidative phosphorylation process (Zhong et al., 2023).

KEGG pathway analysis showed that hypermethylated circRNAs were enriched in the RNA degradation pathway, adherens junction pathway, and hedgehog signaling pathway (Figures 3A, B). The hedgehog (Hh) signaling pathway, a highly conserved evolutionary pathway that is vital during embryonic development as well as cell growth, differentiation, and tissue homeostasis (Jing et al., 2023). Dambergs et al. (Dambergs et al., 2021; Dambergs et al., 2023) found that the Hh signaling pathway was upregulated in both pediatric and adult cholesteatoma, which might play a major role in the hyper-proliferation of cholesteatoma keratinocytes. Importantly, circRNA has been implicated in regulating Hh signaling pathway in multiple cancers, including medulloblastoma (Azatyan et al., 2021), hepatocellular carcinoma (Zhang et al., 2023), and endometrial cancer (Chan and Chen, 2022). The association of Hh with cholesteatoma and the regulatory role of circRNA suggest that m6A hypermethylation in circRNA may play a role in Hh pathway-mediated cholesteatoma pathogenesis.

KEGG analysis in this study found hypomethylated circRNAs to be enriched in the PPAR signaling pathway (Figures 3C, D). PPARs are ligand-activated transcription factors comprised of three different isoforms: PPAR-α, PPAR-β/δ and PPAR-γ. PPARs regulate gene transcription by binding to gene promotors or enhancers, and play an important role in lipid metabolism, glucose homeostasis, cell proliferation, and carcinogenesis (Berger and Moller, 2002). PPAR isoforms are the molecular targets of several lipid lowering and antidiabetic drugs (Berger and Moller, 2002; Atanasov et al., 2013). Previously, Zhang et al. (Zhang et al., 2020). showed PPAR-β/δ isoform was upregulated in human surgical specimens of cholesteatoma which leads to proliferation of cholesteatoma keratinocytes. Hwang et al. (Hwang et al., 2006) also reported that cholesteatoma epithelial cells expressed higher levels of PPAR-γ than normal external auditory canal skin. circRNA has been shown to regulate miRNA, normalizing PPAR and inhibiting hepatocellular steatosis (Guo et al., 2018), suggesting that circRNA may regulate transcription factors and other non-coding RNAs in cholesteatoma pathogenesis.

Middle ear cholesteatoma manifests as excessive squamous epithelial cells, a feature likely related to its disease pathogenesis and progression. Consequently, our investigation focused on identifying specific circRNA molecules associated with proliferation among numerous circRNAs exhibiting different m6A modification profiles. Because these circRNAs have yet to be investigated in the context of middle ear cholesteatoma, their profiles can be leveraged in exploring targets in cholesteatoma treatment. Five circRNAs underwent MeRIP-qPCR: hsa_circRNA_061554, hsa_circRNA_001454, hsa_circRNA_031526, hsa_circRNA_100833, and hsa_circRNA_022382. The results of this validation were consistent with the findings from the microarray analyses, providing evidence that m6A-modified circRNAs play a significant role in the pathogenesis of middle ear cholesteatoma.

Among the circRNAs explored through MeRIP-qPCR (Figure 4), hsa_circRNA_100833 (identified as circFADS2) has been previously proposed as contributing to disease progression. In nonsmall cell lung cancer, circFADS2 has been shown to act as a sponge for miR-498 by binding the target microRNA and inhibiting its activity (Kristensen et al., 2022). This interaction is correlated with poor differentiation, advanced TNM stage, and poor overall patient survival (Zhao et al., 2018). In another study, circFADS2 is under-expressed in sepsis and may protect lung cells from LPS-induced apoptosis by downregulating miR-133a (Niu et al., 2022).

Additionally, hsa_circRNA_001454 and hsa_circRNA_022382 have been shown to be downregulated in Graves’ disease (Sun et al., 2020), an autoimmune disease, and oral squamous cell carcinoma (Zhao et al., 2022), respectively. The pathogenesis of both conditions aligns with proposed etiologies of acquired middle ear cholesteatoma. One proposed theory for cholesteatoma explains that an excessive immune response, as seen in Grave’s, causes the hyperproliferation of the middle ear mucosal lining. Another theory posits that basal cells of the tympanic membrane proliferate and traverse the basement membrane into the middle ear. Similarly, hyperplasia contributes to oral squamous cell carcinoma progression. While the exact regulatory roles of hsa_circRNA_001454 and hsa_circRNA_022382 need to be studied, it is possible that both circRNAs play a role in cholesteatoma pathogenesis.

To our knowledge, we are the first to investigate circRNA m6A methylation modification in a standardized cohort of patients with middle ear cholesteatoma. Nonetheless, the present study had several limitations. The specific functions and downstream targets of the m6A enzymes in this study remain unclear, despite our correlation analysis revealing numerous potential targets. Further studies should focus on identifying the specific targets of m6A regulators and elucidating the exact m6A methylation mechanisms involved in the pathogenesis of middle ear cholesteatoma. This can be achieved by conducting experiments in cell and animal models, such as knocking out or overexpressing specific genes.

## 5 Conclusion

In conclusion, our study represents the first investigation into the m6A methylation alterations of circRNAs in middle ear cholesteatoma, shedding light on previously unexplored molecular mechanisms underlying this condition. By studying into the intricate interplay between m6A methylation modification and circRNAs, we have uncovered potential biological functions and pathways implicated in the pathogenesis of middle ear cholesteatoma. Importantly, our findings not only contribute to a deeper understanding of the molecular processes driving cholesteatoma development and progression but also pave the way for the identification of novel therapeutic targets. Moving forward, further investigations, particularly focused on functional studies of m6A methylation of circRNAs, will be crucial for unraveling the precise molecular intricacies underlying middle ear cholesteatoma pathogenesis.

## Data Availability

The datasets presented in this study can be found in online repositories. The names of the repository/repositories and accession number(s) can be found in the article/Supplementary Material.

## Abbreviations

BP, biological processes; CC, cellular components; ceRNAs, competitive endogenous RNAs; GO, Gene Ontology; IP, immunoprecipitation; KEGG, Kyoto Encyclopedia of Genes and Genomes; circRNA, circular RNA; m6A, N6-methyladenosine; MeRIP-qPCR, Methylated RNA immunoprecipitation combined with quantitative real-time PCR; MF, molecular functions; miRNAs, microRNAs; MREs, microRNA response elements; ncRNAs, non-coding RNAs.
