# Supplementary material for: N6-methyladenosine methylation analysis of circRNAs in acquired middle ear cholesteatoma
Source: Front Genet. 2024 Jun 24;15:1396720. doi: 10.3389/fgene.2024.1396720 (PMC11229040; doi:10.3389/fgene.2024.1396720)
Supplement: Supplementary file 1 [file Table1.DOCX]

**Figure S1.** The flow chart of our research study. GO: Gene Ontology; KEGG: Kyoto Encyclopedia of Genes and Genomes


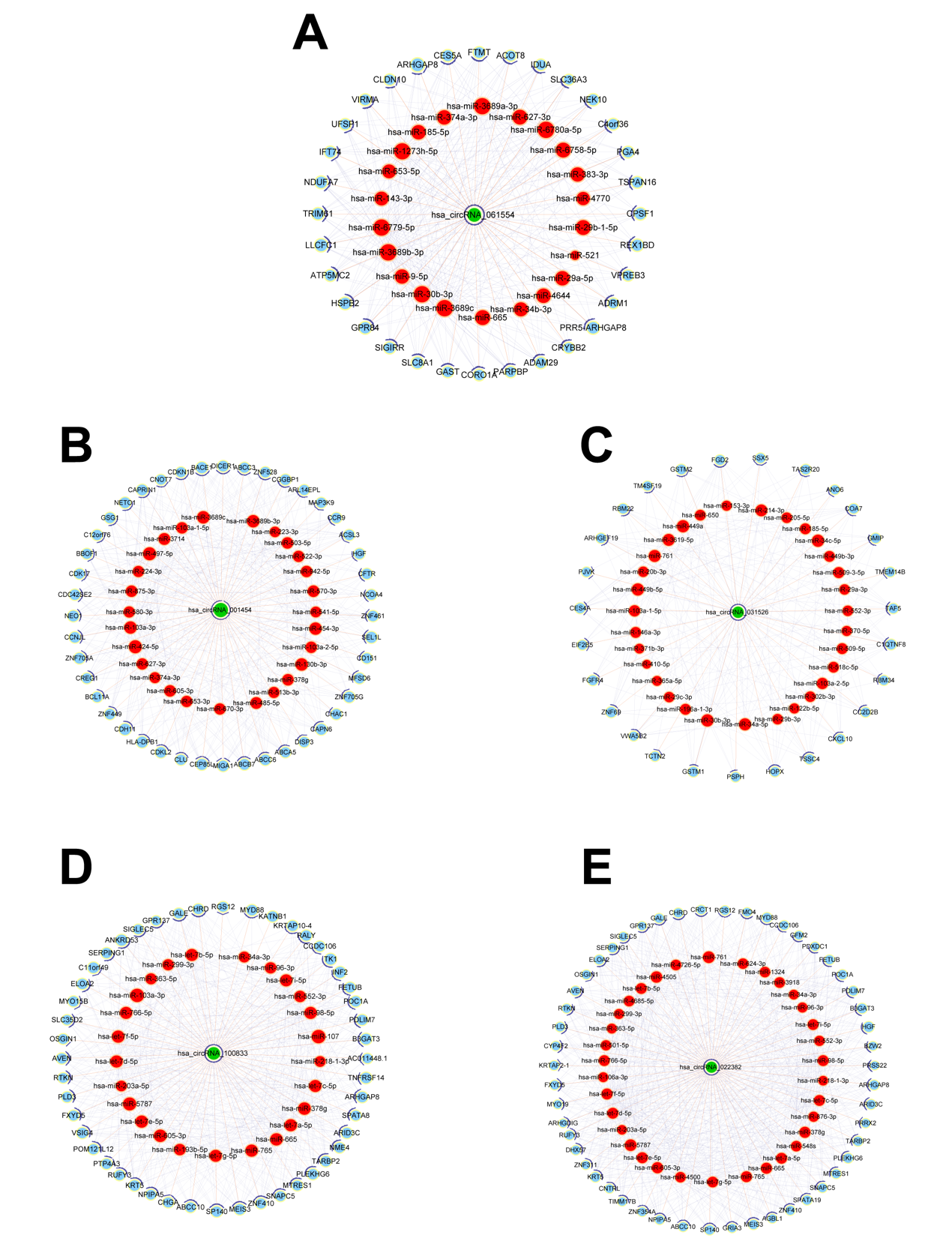


**Figure S2.** The circRNA-miRNA-mRNA network for the five validated m6A-modified circRNAs separately. In the network, circRNAs, microRNAs, and protein coding RNAs are represented by nodes colored in green, red, light blue respectively.
